# A Prospective Study of Renal Blood Flow during Retrograde Intrarenal Surgery

**DOI:** 10.3390/jcm12083030

**Published:** 2023-04-21

**Authors:** Krzysztof Balawender

**Affiliations:** 1 Clinical Department of Urology and Urological Oncology, Municipal Hospital in Rzeszow, 35-241 Rzeszow, Poland; balwender82@gmail.com; Tel.: +48-178-518-906; 2 Department of Normal and Clinical Anatomy, Institute of Medical Sciences, Medical College, Rzeszow University, 35-301 Rzeszow, Poland

**Keywords:** renal blood flow, Doppler ultrasonography, ureteroscopy

## Abstract

(I) Introduction: The use of Doppler ultrasound allows us to indirectly assess the effect of increased intrarenal pressure on renal blood flow during retrograde intrarenal surgery (RIRS). On the basis of vascular flow spectra from selected blood vessels in the kidney, it is possible to determine Doppler parameters that reflect the renal perfusion status, which indirectly shows the degree of vasoconstriction and reflects the resistance of kidney tissue. (II) Materials and methods: A total of 56 patients were included in the study. The study assessed the changes of three Doppler parameters of intrarenal blood flow: resistive index-RI, pulsatility index-PI, and acceleration time-AT in the ipsilateral and contralateral kidneys during RIRS. The effects of mean stone volume, energy used, and pre-stenting were examined as predictors and calculated at two time intervals. (III) Results: The mean value of RI and PI was significantly higher in the ipsilateral kidney than in the contralateral kidney just after RIRS. The mean value of the acceleration time was not significantly different before and after RIRS. The values of all three parameters 24 h after the procedure were comparable to their values immediately after the RIRS. The size of the stone exposed to laser lithotripsy, the value of the energy used, and pre-stenting are not factors that significantly influence Doppler parameters during RIRS. (IV) Conclusions: The significant increase in RI and PI after RIRS in the ipsilateral kidney suggests a vasoconstriction of the interlobar arteries generated by increased intrarenal pressure during the procedure.

## 1. Introduction

Retrograde intrarenal surgery (RIRS) represents an endourological procedure commonly used in the treatment of kidney stones. A significant advantage of RIRS is its minimally invasive nature and its high SFR (stone free rate) [1]. The procedure is dedicated to the treatment of kidney stones smaller than 20 mm, but current studies also indicate that it can be a treatment option in stones larger than 20 mm [2]. In a recent study comparing the effectiveness of RIRS with percutaneous nephrolithotomy of stones 2–4 cm in size, the cumulative SFR for RIRS was 73% [3]. RIRS is considered a safe procedure for the patient, but it is not without risk of kidney damage primarily due to the increased intrarenal pressure mechanism during the procedure. Normal intrarenal pressure ranges from 0 to 5 cm H_2_O and does not exceed 20 cm H_2_O [4,5,6]. The risk of increased intrarenal pressure is due to the use of an irrigation system, which is necessary to perform the procedure with good visibility during laser lithotripsy. Such systems can cause high intrarenal pressures, even above 40 cm of H_2_O (which can be easily reached using a standard manual pumping irrigation) and can cause pyelovenous and pyelolymphatic backflow [7,8]. These increases in intrarenal pressure are related to infectious and hemorrhagic complications, as well as kidney damage [5,9]. The implementation of intrarenal pressure monitoring systems into daily practice is an extremely important challenge in modern endourology Notwithstanding the first studies published with positive feedback on the use of pressure monitoring systems during RIRS, e.g., Pressure Wire system (0.014″, St. Jude Medical, Little Canada, MN, USA), which is now dedicated generally to cardiac procedures, it is still an experimental method [6,10]. Doppler ultrasound is a readily available and non-invasive method of assessing renal blood flow. On the basis of vascular flow spectra from selected blood vessels in the kidney, it is possible to determine Doppler parameters that reflect the renal perfusion status, which indirectly shows the degree of vasoconstriction and reflect the resistance of kidney tissue. The study assessed the variabilities of three Doppler parameters of renal blood flow: resistive index-RI, pulsatility index-PI, and acceleration time-AT.

To my knowledge, this study reports, for the first time in the literature, the results of changes in Doppler renal flow parameters of patients undergoing RIRS at three time points (before the procedure, immediately after the procedure, and 24 h after RIRS); it also presents the relationship of changes in Doppler parameters to the size of the stones and the power generated by the holmium laser during the lithotripsy procedure. An additional aspect of the study is the evaluation of the effect of pre-stenting (insertion of a double J stent) before RIRS on the values of renal flow parameters after the RIRS procedure. The purpose of this study was to determine selected Doppler parameters of renal blood flow in patients with kidney stones treated with RIRS, and to analyze their usability for the assessment of renal perfusion impairment after retrograde intrarenal surgery.

## 2. Materials and Methods

### 2.1. Materials

The Bioethics Committee of Rzeszów University approved the study protocol (2022/040). All procedures performed in studies involving human participants were in accordance with the ethical standards of the institutional and national research committee and with the Declaration of Helsinki of 1964 and its subsequent amendments or comparable ethical standards. Written informed consent was obtained from all participants. Patients who underwent RIRS for renal stones were prospectively included in the study. The study began in May 2022 and ended in September 2022. The inclusion criteria were patients over 18 years of age and patients with renal stones suitable for RIRS treatment. Exclusion criteria were the presence of contralateral renal stones, bleeding disorders, ispi- or contralateral hydronephrosis, renal anomalies, renal failure, a solitary kidney, diabetes mellitus, and hypertension, as well as a significant degree of obesity in the patient, due to the major difficulties in precisely visualizing the ultrasound Doppler flow spectrum in the interlobar vessels. Patients who had a history of renal surgery or ESWL were also excluded from the study. Furthermore, patients receiving treatments (e.g., alpha-blockers, beta-blockers, diuretics, calcium channel blockers, or antidepressants) that could affect the vascular system were excluded. 

The clinical and demographic properties of the patients, including age, sex, body mass index, mean stone volume, and energy used, were collected. During preoperative evaluation, all patients underwent a detailed anamnesis, physical examination, and preoperative radiological evaluation using noncontrast computed tomography, as well as routine laboratory studies (serum creatinine, urinalysis, and urine culture). Positive cultures were determined according to an antibiogram, and surgery was performed under sterile urine. The volume of the stone was calculated according to the formula of Sorokin et al. (A × B × C × 0.524) [11]. 

Doppler assessment of intrarenal vessels was performed using bk 3000 ultrasound scanner model (BK Medical). Doppler studies were performed using the pulse wave method with a convex transducer. The Doppler angle in all measurements was less than 60°. The flow spectrum of the renal interlobar arteries was assessed in the ipsilateral and contralateral kidneys. Each kidney was assessed in three projections: upper pole, mid segment, and lower pole. Examinations were performed at fixed intervals before surgery, immediately after surgery, and 24 h after surgery. The following parameters were calculated from the vascular flow spectrum of the visualized interlobar arteries: renal resistive index (RI), pulsatility index (PI), and acceleration time (AT). RI and PI were calculated on the basis of the values of PSV (peak systolic velocity) and EDV (end-diastolic velocity). RI uses the formula (PSV − EDV)/PSV, whereas PI uses the formula [(PSV − EDV)/(PSV + EDV)/2] [12,13]. Acceleration time (AT) of the interlobar arteries is calculated by identifying the point of systolic upstroke and the first (early) systolic peak. The values of the above parameters were automatically calculated from the flow spectrum recordings of the interlobar arteries of the kidney using ultrasound software. The numerical values of the individual parameters used in the statistical analysis were the arithmetic mean of the three results (assessed in three projections for each kidney in each time period). The surgical procedure was performed by an experienced surgeon under general anesthesia. The surgical procedure began with standard cystoscopy and retrograde pyelography, with a 0.035-inch safety guidewire (BiWire; Cook Medical; Bloomington, IN, USA). A 12 Fr. ureteral access sheath (Flexor; Cook Medical; Bloomington, IN, USA), was inserted over the guidewire and placed just 1 cm below the ureteropelvic junction. To standardize the study population, all surgeries performed without the use of a ureteral access sheath were excluded from the study. The bladder was drained using a 10 Fr. feeding tube during the procedure. A flexible ureteroscope (Pusen; PU3022A) was inserted through the ureteral access sheath. A holmium:YAG laser (Quanta System Cyber Ho 60W; Samarate, Italy) with a 272 mm laser fiber (Quanta System; Samarate, Italy) was used to fragment the stones. Constant gravity-based irrigation was used, with a height of 50 cm above the patient, and a hand-pumping system was used if necessary. Laser energy and pulse frequency were varied according to the stone burden and the surgeon’s preference. A 4.7 Fr. double J stent was inserted into the urinary system and left in place for 2–3 weeks.

### 2.2. Methodology

The significance level of the statistical tests in the present analysis was set at α = 0.05. The distribution measures of the central tendency/dispersion for the numerical variables were expressed in terms of median Mdn (Q1–Q3). The Mann–Whitney *U* test was used to compare the means of two independent groups with non-normal distributions.

#### 2.2.1. Regression Analysis

Hypotheses were tested based on the multilevel variant of the simple change score model according to Formulae (1)–(3):

y_it_ ~ N (μ_it_, σ_ε_), (1)

μ_it_ = β_0_ + β_1_ kidney_it_ + β_2_ time_it_ + β_3_ kidney × _it_time_it_ + β_4_ predictor_it_ + u_0i_, (2)

u_0i_ ~ N (0, σ_0_), (3)

where the outcome variable (focal) y was measured at the two time levels (before RIRS and just after RIRS; just after RIRS and 24 h after RIRS); β_0_ (β-non-standardized regression coefficient) was the population mean for the change for the first time point and the reference groups for noncontinuous predictors and the value 0 for continuous; β_1_ was the difference at the population level of the ipsilateral kidney compared to the contralateral (further kidney side) at the first time point; β_2_ was the change over time for the ipsilateral kidney; β_3_ was the time-by-kidney interaction, which also corresponded to the average kidney effect in the sample; β_4_ was the change over time for the selected predictor.

Since only two time points were considered at once, we cannot have both random intercepts and time courses. However, the model does account for the variation at the participant level around the grand mean of baseline u_0i_, which was modelled as normally distributed with a mean of zero and a standard deviation σ_0_ estimated as part of the model. Such a model structure allowed for potential correlation between kidneys in the same individual.

The resistive index, pulsatility index, and acceleration time were included in the form of the focal variable y. The effects of mean stone volume, energy used, and pre-stenting were examined as predictors. To make the model robust, only one of the predictors mentioned above was simultaneously considered in the model. Furthermore, the effects of the variables were calculated at two time intervals: (a) before RIRS–0 h after RIRS; (b) 0 h after RIRS–24 h after RIRS. The effects of the above variables were calculated on the basis of Formulae (1)–(3).

The mixed linear model was estimated using a restricted maximum likelihood (REML) and nloptwrap optimizer. The 95% confidence intervals (CIs) and p-values were calculated using a Wald t-distribution approximation. The coding of dichotomous variables was performed with the dummy method (with first category as reference one).

The proportion of variance in the response variable that was explained (i.e., predicted) by the predictors was reported by the marginal determination coefficient R^2^_conditional_ (the total model, i.e., both fixed and random effects) and R^2^_marginal_ (for fixed effects only). The R^2^ marginal determination coefficient magnitude was interpreted by Cohen’s convention [14]. The proportion of variance explained by the grouping structure in the population was estimated by intraclass correlation coefficient (ICC) interpreted by the Koo convention [15].

The normality of studentized residuals of fitting models was tested by the Shapiro–Wilk test and by Q–Q plots. To evaluate the performance of the model, the posterior predictive test, homogeneity of variance, collinearity, and linearity of the model were examined.

#### 2.2.2. Statistical Environment

Analyses were performed using the R Statistical language (version 4.1.1; R Core Team, 2021, R Foundation for Statistical Computing, Vienna, Austria) on Windows 10 × 64 (build 19044), using the packages lme4 (version 1.1.27.1), Matrix (version 1.5.1), effectsize (version 0.8.2), Rcpp (version 1.0.7), purrr (version 0.3.4), rstatix (version 0.7.1), emmeans (version 1.8.2), sjPlot (version 2.8.11), performance (version 0.10.0), report (version 0.5.1.3), psych (version 2.1.6), ggplot2 (version 3.4.0), tidyverse (version 1.3.2), readxl (version 1.3.1), dplyr (version 1.0.10), tidyr (version 1.2.0), and readr (version 2.1.3).

### 2.3. Characteristic of the Sample

The sample of 56 subjects was analyzed, including 22 (40.0%) women and 33 (60.0%) men. Additional sociodemographic data can be found in Table 1. The characteristics of the clinical data analyzed with and without repeated measures are listed in Table 2 and Table 3.

## 3. Results

All models were fitted based on a sample with N_obs_ = 220, N_id_ (sample size of individuals) = 55.

### 3.1. Regression Models for the Time-Point Predictor before RIRS–0 h after RIRS (Mod1–Mod9)

#### 3.1.1. Resistive Index as Focal Variable

**Figure 1:**
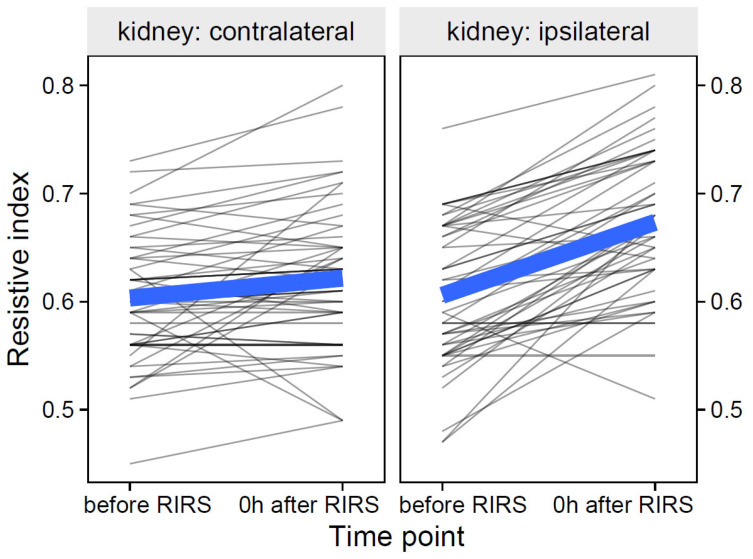
The effect of kidney side on resistive index over time (the thin, semitransparent black lines in the background are the individual-level data. The bold blue lines in the foreground are the group averages). The figure shows that the effects of the kidney side on the focal variable of the resistance index were obviously different over time. The inclusion of the effects of mean stone volume, energy used, pre-stenting, and kidney side at separate time points is presented in Section 3.1.1.1–Section 3.1.1.3.

Participant-level data of the resistive index and their group means over time are shown in Figure 1.

##### 3.1.1.1. Estimation of the Effects of the Volume of the Mean Stone, Kidney Side and Time on the Resistive Index (Mod1)

**Figure 2:**
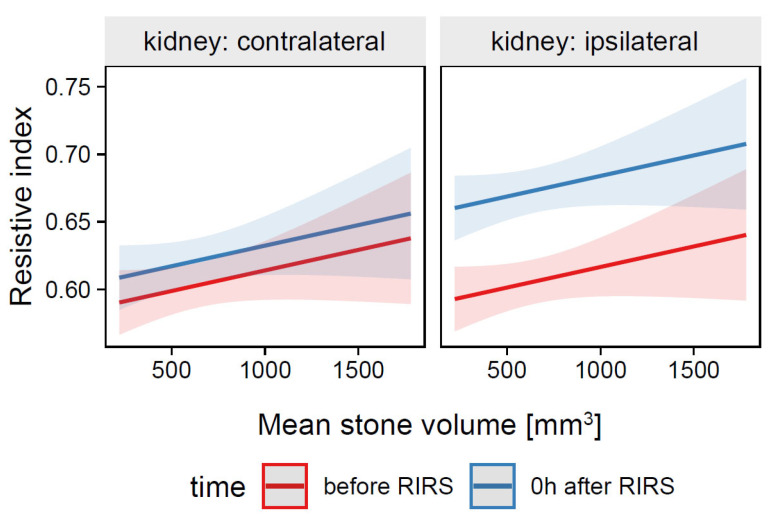
Predicted values (marginal effects) of resistive index by the Mod1 regression model for mean stone volume, time, and kidney side terms.

The total explanatory power of the Mod1 model was substantial R^2^_conditional_ = 0.71 and the part related to the fixed effects alone R^2^_marginal_ was 0.19, while the ICC (intraclass correlation coefficient) was moderate (0.64). The intercept of the model, corresponding to the time before RIRS, contralateral kidney, and mean stone volume = 0 mm^3^, was at β_0_ *=* 0.58 (95% CI [0.55, 0.61], *t*(213) = 36.90, *p* < 0.001). There were no significant differences in the resistive index between the ipsilateral and contralateral kidneys before RIRS, β_1_ *=* 0.00, 95% CI [−0.01, 0.02], *p* = 0.721. The mean value of the resistive index was significantly higher 0 h after RIRS than before RIRS, β_2_ = 0.02, *95%* CI [0.00, 0.03], *p* = 0.010. There was no significant effect of the mean stone volume on resistive index, β_4_ = 0.00, 95% CI [0.0, 0.00], *p* = 0.142. The resistive index was significantly higher in the ipsilateral kidney than in the contralateral kidney at time 0 h after RIRS, β_3_ *=* 0.05, 95% CI [0.03, 0.07], *p* < 0.001. For prediction plot, see Figure 2.

##### 3.1.1.2. Estimation of the Effects of Energy Used, Kidney Side, and Time on the Resistive Index (Mod2)

**Figure 3:**
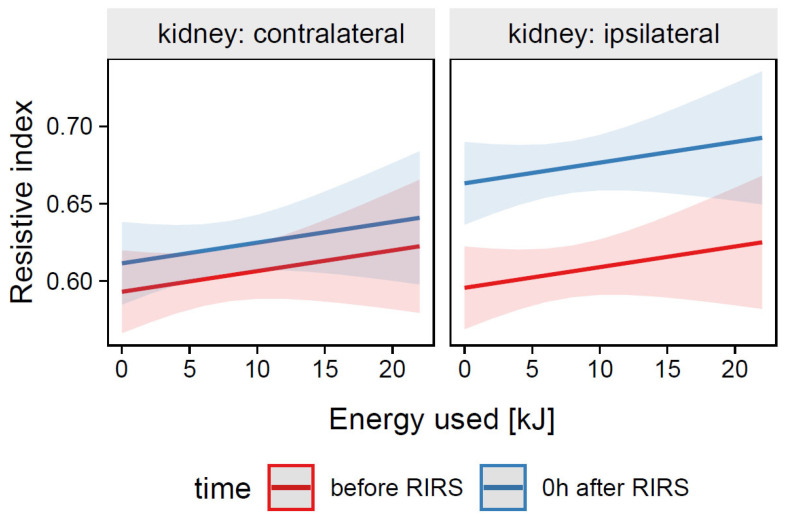
Predicted values (marginal effects) of resistive index by the Mod2 regression model for energy used, time, and kidney side terms.

The total explanatory power of the Mod2 model was substantial R^2^_conditional_ = 0.71 and the part related to fixed effects alone R^2^_marginal_ was 0.18, while the ICC was moderate (0.65). The model intercept corresponding to the time before RIRS, contralateral kidney, and energy used = 0 kJ was at β_0_ *=* 0.59 (95% CI [0.57, 0.62], *t*(213) = 43.54, *p* < 0.001). There was no significant effect of energy used on resistive index, β_4_ *=* 0.00, 95% CI [0.00, 0.00], *p* = 0.343. For prediction plot, see Figure 3.

##### 3.1.1.3. Estimation of the Effects of Pre-Stenting, Kidney Side, and Time on the Resistive Index (Mod3)

**Figure 4:**
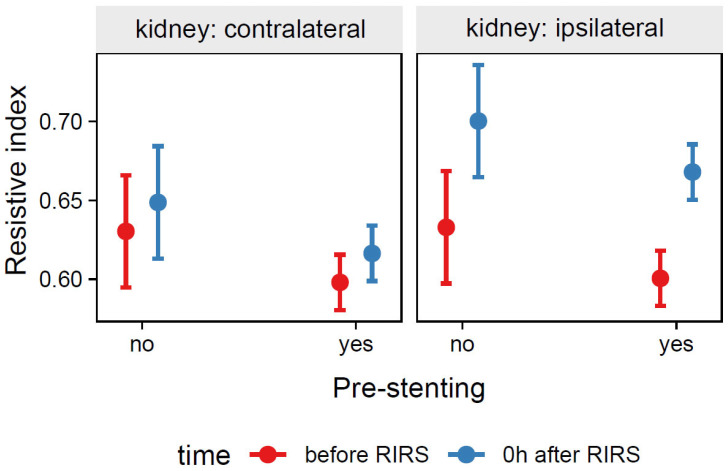
Predicted values (marginal effects) of resistive index by the Mod3 regression model for pre-stenting, time, and kidney side terms.

The total explanatory power of the Mod3 model was substantial R^2^_conditional_ = 0.71 and the part related to the fixed effects alone R^2^_marginal_ was 0.20, while the ICC was moderate (0.64). The intercept of the model corresponding to the time before RIRS, contralateral kidney side, and the lack of pre-stenting was at β_0_ = 0.63 (95% CI [0.59, 0.67], *t*(213) = 34.63, *p* < 0.001). The pre-stenting procedure had no significant effect on the resistive index compared to non-pre-stenting before RIRS, β_4_ *=* −0.03, 95% CI [−0.07, 0.01], *p* = 0.096. For prediction plot, see Figure 4.

#### 3.1.2. Pulsatility Index as Focal Variable

**Figure 5:**
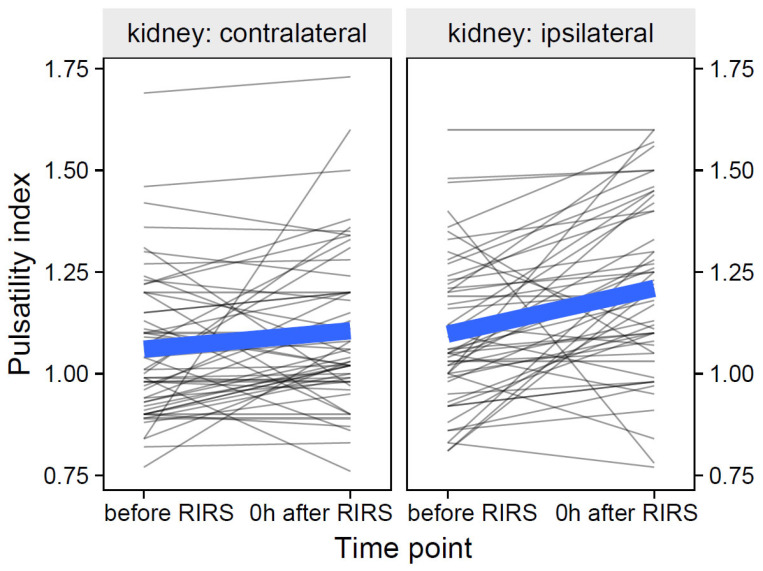
The effect of kidney side on the pulsatility index over time (the thin, semitransparent black lines in the background are the individual-level data. The bold blue lines in the foreground are the group averages). The figure shows that the effects of the kidney side on the focal variable of the pulsatility index were quite different over time. The inclusion of the effects of mean stone volume, energy used, pre-stenting, and kidney side at separate time points is presented in Section 3.1.2.1–Section 3.1.2.3.

The participant-level data of the pulsatility index and its group means over time are shown in Figure 5.

##### 3.1.2.1. Estimation of the Effects of Mean Stone Volume, Kidney Side, and Time on Pulsatility Index (Mod4)

**Figure 6:**
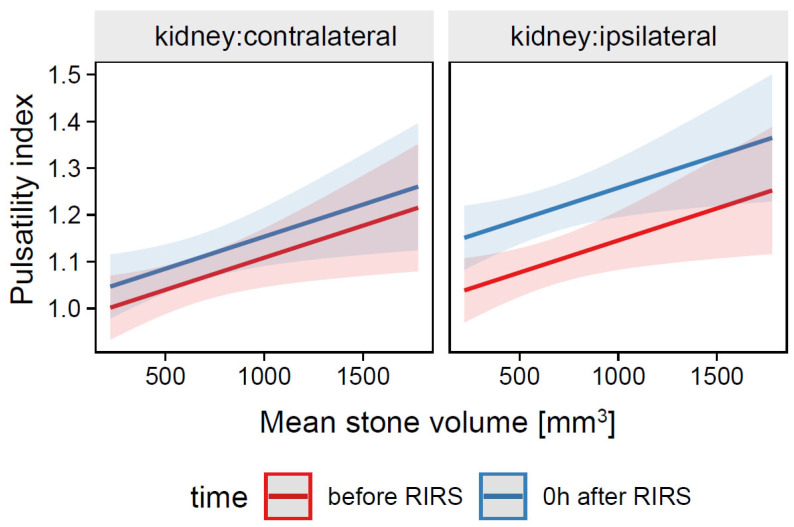
Predicted values (marginal effects) of pulsatility index by the Mod4 regression model for mean stone volume, time and kidney side terms.

The total explanatory power of the Mod4 model was substantial R^2^_conditional_ = 0.58 and the part related to fixed effects alone R^2^_marginal_ was 0.14, while the ICC was moderate (0.51). The model intercept corresponding to the time before RIRS, contralateral kidney, and mean stone volume = 0 mm^3^ was at β_0_ *=* 0.97 (95% CI [0.88, 1.06], *t*(213) = 21.73, *p* <0.001). There were no significant differences in the pulsatility index between the ipsilateral and contralateral kidneys before RIRS, β_1_ = 0.04, 95% CI [−0.01, 0.09], *p* = 0.138. The pulsatility index of the ipsilateral kidney was significantly higher than that of the contralateral kidney at time 0 h after RIRS, β_3_ = 0.10, 95% CI [0.07, 0.14], *p* < 0.001. The change in pulsatility index in the contralateral kidney just after RIRS compared with before RIRS was 0.04 (1.06 before RIRS and 1.10 after RIRS) and not significant, *p* = 0.218, whereas the change in pulsatility index in the ipsilateral kidney was 0.11 (1.10 before RIRS, 1.21 after RIRS) and statistically significant, *p* < 0.001. For prediction plot, see Figure 6.

##### 3.1.2.2. Estimation of the Effects of Energy Used, Kidney Side, and Time on the Resistive Index (Mod5)

**Figure 7:**
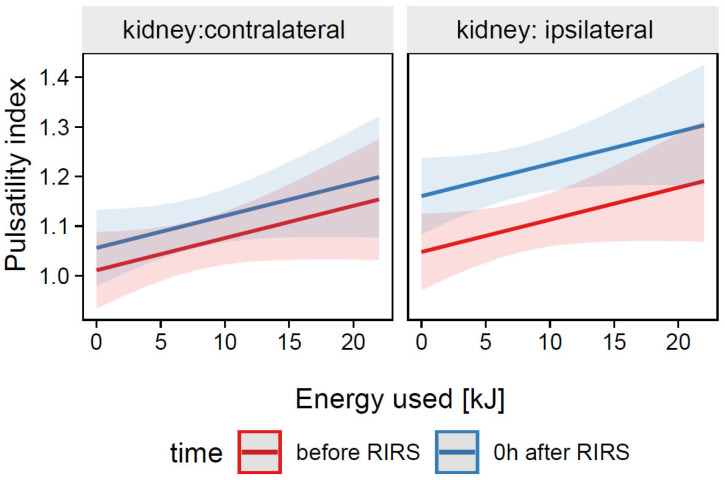
Predicted values (marginal effects) of pulsatility index by the Mod5 regression model for energy used, time and kidney side terms.

The total explanatory power of the Mod5 model was substantial R^2^_conditional_ = 0.58 and the part related to fixed effects alone R^2^_marginal_ was 0.11, while the ICC was moderate (0.53). The model intercept corresponding to the time before RIRS, contralateral kidney, and energy used = 0 kJ was at β_0_ *=* 1.01 (95% CI [0.93, 1.09], *t*(213) = 25.74, *p* < 0.001. The mean value of the pulsatility index was not significantly different between 0h after RIRS and before *RIRS*, β_2_ = 0.05, 95% CI [−0.00, 0.09], *p* = 0.070 There was also no significance effect of energy used on pulsatility index, β_4_ = 0.01, 95% CI [0.0, 0.01], *p* = 0.101. For prediction plot, see Figure 7.

##### 3.1.2.3. Estimation of the Effects of Pre-Stenting, Kidney Side, and Time on Resistive Index (Mod6)

**Figure 8:**
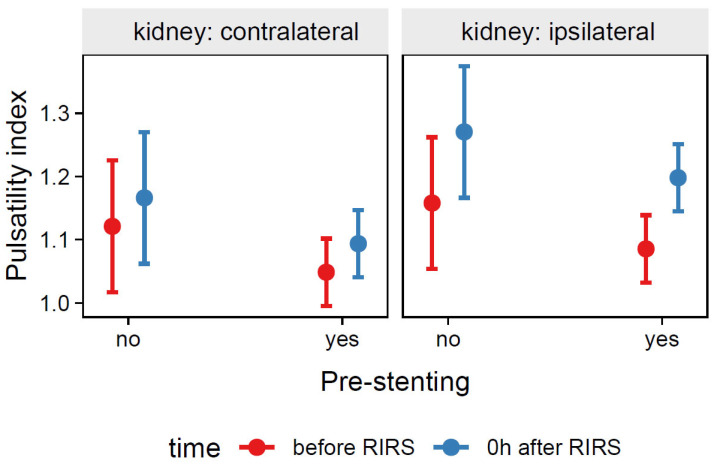
Predicted values (marginal effects) of resistive index by the Mod6 regression model for pre-stenting, time and kidney side terms.

The total explanatory power of the Mod6 model was substantial R^2^_conditional_ = 0.58 and the part related to fixed effects alone R^2^_marginal_ was 0.10, while the ICC was moderate (0.53). The model intercept corresponding to the time before RIRS, contralateral kidney, and lack of pre-stenting was at β_0_ = 1.12 (95% CI [1.02, 1.23], *t*(213) = 34.63, *p* < 0.001). The pre-stenting procedure had no significant effect on the pulsatility index compared with non-pre-stenting before RIRS, β_4_ = −0.07, 95% CI [−0.18, 0.04], *p* = 0.194. For prediction plot, see Figure 8.

#### 3.1.3. Acceleration Time as Focal Variable

**Figure 9:**
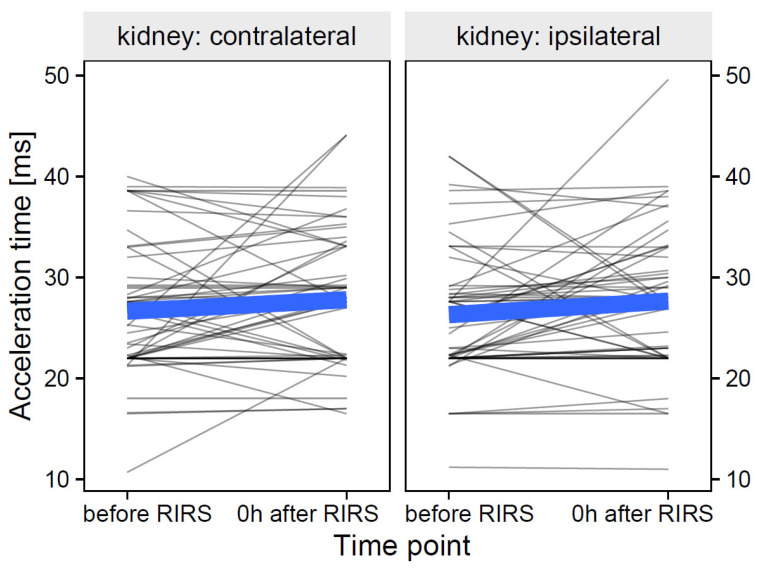
The effect of kidney side on acceleration time over time (the thin, semi-transparent black lines in the background are the individual-level data. The bold blue lines in the foreground are the group averages). The figure shows that the effects of the kidney side on the focal variable of the acceleration time did not differ over time. The inclusion of the effects of mean stone volume, energy used, pre-stenting, and kidney side at separate time points is presented in Section 3.1.3.1–Section 3.1.3.3.

The data of acceleration time at the participant level and their group means over time are shown in Figure 9.

##### 3.1.3.1. Estimation of the Effects of Mean Stone Volume, Kidney Side, and Time on Acceleration Time (Mod7)

The total explanatory power of the Mod7 model was substantial R^2^_conditional_ = 0.52 and the part related to fixed effects alone R^2^_marginal_ was 0.02, while the ICC was moderate (0.51). The model intercept corresponding to the time before RIRS, contralateral kidney, and mean stone volume = 0 mm^3^ was at β_0_ = 25.48 (95% CI [22.30, 28.66], *t*(213) = 15.80, *p* < 0.001). There were no significant differences in the acceleration time between the ipsilateral and contralateral kidneys prior to RIRS, β_1_ = −0.35, 95% CI [−2.11, 1.42], *p* = 0.699. The mean value of the acceleration time was not significantly different between 0 h after RIRS and before RIRS, β_2_ = 1.14, 95% CI [−0.63, 2.91], *p* = 0.206. There was also no significant effect of the mean stone volume on acceleration time, β_4_ = 0.00, 95% CI [0.0, 0.01], *p* = 0.380. Acceleration time was not significantly different between the ipsilateral and contralateral kidneys at time after RIRS, β_3_ = 0.21, 95% CI [−2.29, 2.71], *p* = 0.868.

##### 3.1.3.2. Estimation of the Effects of Energy used, Kidney Side, and Time on Acceleration Time (Mod8)

The total explanatory power of the Mod8 model was substantial R^2^_conditional_ = 0.52 and the part related to fixed effects alone R^2^_marginal_ was 0.01, while the ICC was moderate (0.51). The model intercept corresponding to the time before RIRS, contralateral kidney, and energy used = 0 kJ was at β_0_ = 27.17 (95% CI [24.43, 29.91], *t*(213) = 19.52, *p* < 0.001. There was also no significant effect of the energy used on the acceleration time, β_4_ = −0.07, 95% CI [−0.34, 0.21], *p =* 0.626.

##### 3.1.3.3. Estimation of the Effects of Pre-Stenting, Kidney Side, and Time on Acceleration Time (Mod9)

The total explanatory power of the Mod9 model was substantial R^2^_conditional_ = 0.52 and the part related to fixed effects alone R^2^_marginal_ was 0.02, while the ICC was moderate (0.50). The model intercept corresponding to the time before RIRS, contralateral kidney and lack of pre-stenting was at β_0_ = 24.75 (95% CI [21.12, 28.39], *t*(213) = 13.41, *p* < 0.001. The pre-stenting procedure did not have a significant effect on the acceleration time compared to non-pre-stenting before RIRS, β_4_ = 2.27, 95% CI [−1.53, 6.07], *p* = 0.240.

### 3.2. Regression Models for the Time Point Predictor 0 h after RIRS-24 h after RIRS (Mod10–Mod12)

#### 3.2.1. Resistive Index as Focal Variable

**Figure 10:**
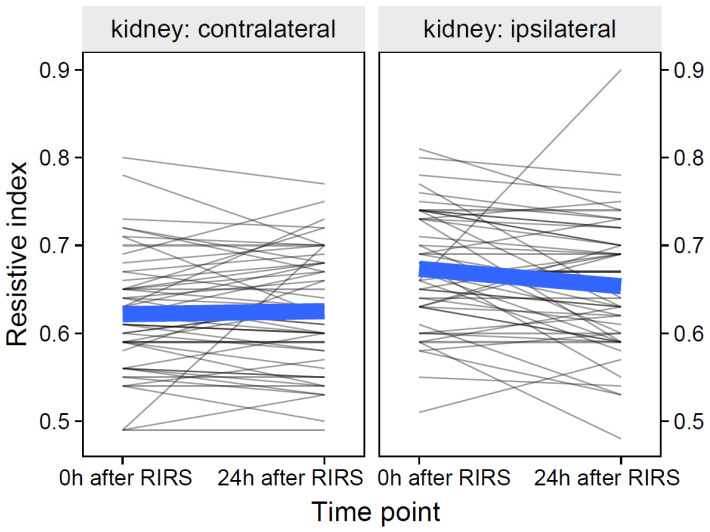
The effect of kidney side on resistive index over time (the thin, semitransparent black lines in the background are the individual-level data. The bold blue lines in the foreground are the group averages). The figure shows that the effects of the kidney side on the focal variable of the resistance index were obviously different over time. The inclusion of the effects of mean stone volume, energy used, pre-stenting, and kidney side at separate time points is presented in Section 3.2.1.1–Section 3.2.1.3.

The data of the resistive index participants and their group means over time are shown in Figure 10.

##### 3.2.1.1. Estimation of the effects of mean stone volume, kidney side, and time on resistive index (Mod10)

The total explanatory power of the Mod10 model was substantial R^2^_conditional_ = 0.65 and the part related to fixed effects alone R^2^_marginal_ was 0.10, while the ICC was moderate (0.61). The model intercept corresponding to the time 0 h after RIRS, contralateral kidney, and mean volume of the stone = 0 mm^3^ was at β_0_ = 0.61 (95% CI [0.57, 0.64], *t*(213) = 35.60, *p* < 0.001). The resistive index of the ipsilateral kidney was significantly higher than that of the contralateral kidney at time 0 h after RIRS, β_1_ = 0.05, 95% CI [0.04, 0.07], *p* < 0.001 The mean value of the resistive index was not significantly different between 0 h after RIRS and 24 h after RIRS, β_2_ = 0.00, 95% CI [−0.01, 0.02], *p* = 0.672.There was no significant effect of the mean stone volume on the resistive index, β_4_ = 0.00, 95% CI [0.0, 0.00], *p* = 0.261.

##### 3.2.1.2. Estimation of the Effects of Energy Used, Kidney Side, and Time on the Resistive Index (Mod11)

The total explanatory power of the Mod11 model was substantial R^2^_conditional_ = 0.65 and the part related to fixed effects alone R^2^_marginal_ was 0.09, while the ICC was moderate (0.61). The intercept of the model corresponding to time 0h after RIRS, contralateral kidney, and energy used = 0 kJ was at β_0_ = 0.61 (95% CI [0.58, 0.64], *t*(213) = 41.97, *p* < 0.001). There was no significant effect of the energy used on the resistive index, β_4_ = 0.00, 95% CI [0.0, 0.00], *p* = 0.484.

##### 3.2.1.3. Estimation of the Effects of Pre-Stenting, Kidney Side, and Time on the Resistive Index (Mod12)

The total explanatory power of the Mod12 model was substantial R^2^_conditional_ = 0.65 and the part related to the fixed effects alone R^2^_marginal_ was 0.11, while the ICC was moderate (0.60). The intercept of the model corresponding to time 0h after RIRS, contralateral kidney, and lack of pre-stenting was at β_0_ *=* 0.65 (95% CI [0.61, 0.69], *t*(213) = 33.19, *p* < 0.001). The pre-stenting procedure did not have a significant effect on the resistive index compared to the non-pre-stenting 0 h after RIRS, β_4_ = −0.03, 95% CI [−0.07, 0.01], *p* = 0.140.

#### 3.2.2. Pulsatility Index as Focal Variable

**Figure 11:**
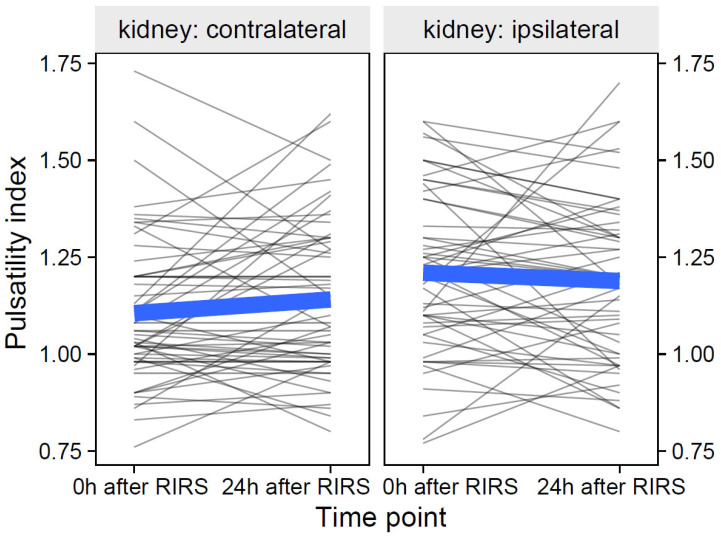
The effect of kidney side on the pulsatility index over time (the thin, semitransparent black lines in the background are the individual-level data. The bold blue lines in the foreground are the group averages). The figure shows that the effects of the kidney side on the focal variable of the pulsatility index were obviously different over time. The inclusion of the effects of mean stone volume, energy used, pre-stenting, and kidney side at separate time points is presented in Section 3.2.2.1–Section 3.2.2.3.

The participant-level data of the pulsatility index and its group means over time are shown in Figure 11.

##### 3.2.2.1. Estimation of the Effects of Mean Stone Volume, Kidney Side, and Time on Pulsatility Index (Mod13)

The total explanatory power of the Mod13 model was substantial R^2^_conditional_ = 0.60 and the part related to fixed effects alone R^2^_marginal_ was 0.07, while the ICC was moderate (0.57). The model intercept corresponding to time 0 h after RIRS, contralateral kidney, and mean stone volume = 0 mm^3^ was at β_0_ = 1.04 (95% CI [0.94, 1.14], *t*(213) = 20.87, *p* < 0.001). The ipsilateral kidney pulsatility index was significantly higher than the contralateral kidney at time 0 h after RIRS, β_1_ = 0.10, 95% CI [0.05, 0.15], *p* < 0.001. The mean value of the pulsatility index was not significantly different between 0 h after RIRS and 24 h after RIRS, β_2_ = 0.04, 95% CI [−0.01, 0.08], *p* = 0.116. There was no significant effect of mean stone volume on the pulsatility index, β_4_ = 0.00, 95% CI [0.0, 0.00], *p* = 0.102. Pulsatility index was not significantly different between ipsilateral and contralateral kidneys at time 24 h after RIRS, β_3_ = −0.06, 95% CI [−0.13, 0.01], *p =* 0.112.

##### 3.2.2.2. Estimation of the Effects of Energy Used, Kidney Side, and Time on the Pulsatility Index (Mod14)

The total explanatory power of the Mod14 model was substantial R^2^_conditional_ = 0.60 and the part related to fixed effects alone R^2^_marginal_ was 0.05, while the ICC was moderate (0.58). The intercept of the model corresponding to time 0 h after RIRS, contralateral kidney, and energy used = 0 kJ was at β_0_ = 1.07 (95% CI [0.99, 0.16], *t*(213) = 24.90, *p* < 0.001). There was no significant effect of the energy used on the pulsatility index, β_4_ = 0.00, 95% CI [0.0, 0.01], *p* = 0.351.

##### 3.2.2.3. Estimation of the Effects of Pre-Stenting, Kidney Side, and Time on the Pulsatility Index (Mod15)

The total explanatory power of the Mod15 model was substantial R^2^_conditional_ = 0.60 and the part related to fixed effects alone R^2^_marginal_ was 0.05, while the ICC was moderate (0.58). The model intercept corresponding to the time 0 h after RIRS, contralateral kidney, and lack of pre-stenting was at β_0_ = 1.15 (95% CI [1.03, 1.26], *t*(213) = 19.71, *p* < 0.001). The pre-stenting procedure had no significant effect on the pulsatility index compared with non-pre-stenting just after RIRS, β_4_ = −0.05, 95% CI [−0.17, 0.07], *p* = 0.398.

#### 3.2.3. Acceleration Time Index as Focal Variable

**Figure 12:**
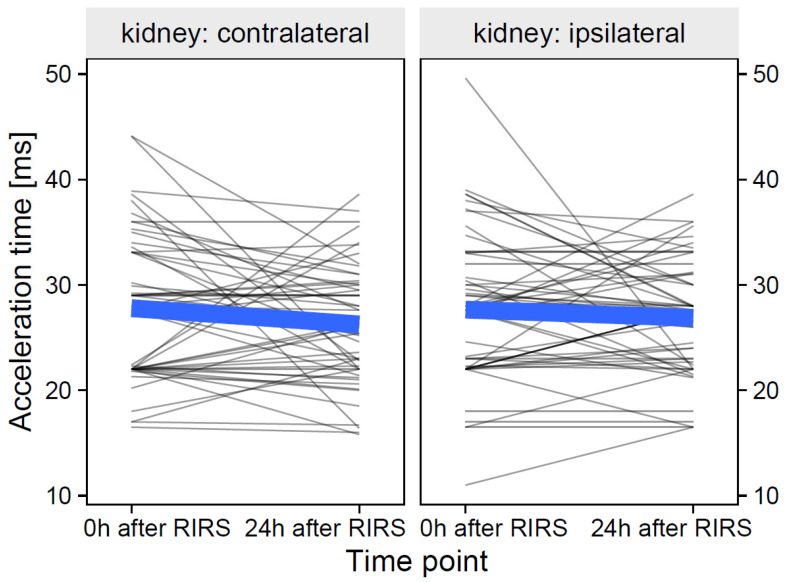
The effect of kidney side on the pulsatility index over time (the thin, semitransparent black lines in the background are the individual-level data. The bold blue lines in the foreground are the group averages). The figure shows that the effects of the kidney side on the acceleration time focal variable were slightly different over time. The inclusion of the effects of mean stone volume, energy used, pre-stenting, and kidney side at separate time points is presented in Section 3.2.3.1–Section 3.2.3.3.

The data of acceleration time at the participant level and their group means over time are shown in Figure 12.

##### 3.2.3.1. Estimation of the Effects of the Volume of the Mean Stone, the Kidney Side and the Time on the Acceleration Time (Mod16)

The total explanatory power of the Mod16 model was substantial R^2^_conditional_ = 0.54 and the part related to fixed effects alone R^2^_marginal_ was 0.01, while the ICC was moderate (0.54). The model intercept corresponding to 0 h after RIRS, contralateral kidney, and mean stone volume = 0 mm^3^ was at β_0_ = 27.00 (95% CI [24.01, 29.99], *t*(213) = 17.82, *p* < 0.001). There were no significant differences in acceleration time between the ipsilateral and contralateral kidneys 0 h after RIRS, β_1_ = −0.14, 95% CI [−1.73, 1.45], *p* = 0.866. The mean value of the acceleration time was not significantly different between 0 h after RIRS and 24 h after RIRS, β_2_ = −1.54, 95% CI [−3.13, 0.05], *p* = 0.057. There was also no significant effect of the mean stone volume on acceleration time, β_4_ = 0.00, 95% CI [0.0, 0.01], *p* = 0.533. Acceleration time was not significantly different between the ipsilateral and contralateral kidneys at time 24 h after RIRS, β_3_ = 0.76, 95% CI [−1.49, 3.01], *p* = 0.505.

##### 3.2.3.2. Estimation of the Effects of Energy Used, Kidney Side, and Time on Acceleration Time (Mod17)

The total explanatory power of the Mod17 model was substantial R^2^_conditional_ = 0.54 and the part related to fixed effects alone R^2^_marginal_ was 0.01, while the ICC was moderate (0.55). The model intercept corresponding to 0 h after RIRS, contralateral kidney, and energy used = 0 kJ was at β_0_ = 28.20 (95% CI [25.63, 30.76], *t*(213) = 21.65, *p* < 0.001). There was also no significant effect of the energy used on the acceleration time, β_4_ = −0.05, 95% CI [−0.31, 0.21], *p =* 0.685.

##### 3.2.3.3. Estimation of the Effects of Pre-Stenting, Kidney Side, and Time on Acceleration Time (Mod18)

The total explanatory power of the Mod18 model was substantial R^2^_conditional_ = 0.54 and the part related to fixed effects alone R^2^_marginal_ was 0.02, while the ICC was moderate (0.53). The model intercept corresponding to 0 h after RIRS, contralateral kidney, and lack of pre-stenting was at β_0_ = 26.25 (95% CI [22.83, 29.67], *t*(213) = 21.65, *p* < 0.001). The pre-stenting procedure had no significant effect on the acceleration time compared to non-pre-stenting 0 h after RIRS, β_4_ = 1.84, 95% CI [−1.74, 5.43], *p* = 0.312.

## 4. Discussion

Retrograde intrarenal surgery (RIRS) is associated with the risk of increased intrapelvic pressure (IRP) due to the necessity of continuous irrigation and manual pumping during the procedure. Increases in intrarenal pressure can lead to pyelorenal backflow during RIRS, which occurs at intrarenal pressures evident after 40 cm of H_2_O [16]. Based on experiments carried out to evaluate IRP in vivo in humans and in vitro in animals or artificial models, depending on the irrigation system used (pump or gravity), the mean IPR was in the range of several dozens of cm H_2_O, while the maximum pressure registered during RIRS often exceeded 100 cm H_2_O [6,10,17]. The high intrarenal pressure generated during RIRS can result in pathophysiological and histological changes in the pyelocalyceal system. Schwalb et al. evaluated renal pressure in vitro using freshly harvested porcine kidneys and proved that increased pressure can cause diffuse denudation and flattening of the calyx urothelium, submucosal oedema, and congestion, which can result in hypoperfusion around treated calyces. It is hypothesized that in the early stages of high intrarenal pressure, bacterial translocation occurs, which, with a high bacterial load, can result in urinary tract infection and sepsis. A late effect of elevated intrarenal pressure increases the risk of columnar metaplasia, subepithelial nests, and perivasculitis, resulting in focal scarring after four to six weeks [13,18]

Currently, we do not have a device that allows continuous monitoring of intrarenal pressure during RIRS, and single reports on the use of probes to measure pressure in the pyelocalyceal system are at an experimental stage [10,19]. Hence, non-invasive evaluation of renal Doppler blood flow parameters provides an indirect assessment of the dynamic changes that are occurring in the kidney during the RIRS procedure. The renal resistive index and the pulsatility index are the most well-known and widely used Doppler parameters of renal blood flow. The parameters are broadly used in the monitoring of vascular and interstitial pathologies in the kidneys and provide information about arterial impedance. RI and PI are indirect markers of the degree of vasoconstriction and reflect tissue resistance to blood flow caused by cellular infiltration, interstitial oedema, increased hydrostatic pressure, and colloid osmotic pressure [13]. The third parameter of renal blood flow assessed in this study was acceleration time (AT). This parameter, similar to RI and PI, is assessed on the basis of flow spectrum in interlobar arteries, and provides an indirect measure of impediment of blood flow into the study area. To my knowledge, this study is the first prospective human study to evaluate three Doppler parameters of changes in renal blood flow after RIRS.

The assessment of Doppler parameters of renal blood flow has been widely used in the transplantology field as a repeatable and noninvasive method to assess the hemodynamics of the transplanted kidney. Doppler parameters calculated from the intrarenal arterial flow spectrum have been used to assess the safety of ESWL procedures. Balawender et al. reported that extracorporeal shock wave lithotripsy (ESWL) of renal stone causes temporary impairment of renal perfusion in both ipsilateral and contralateral kidneys based on RI and PI measurements. The study demonstrated in a group of 42 patients a significant increase in the RI value measured directly after the procedure, based on the blood flow of the interlobar artery in both the kidney subjected to lithotripsy and the contralateral kidney, and a gradual normalization of the RI values in both kidneys 92 h after the procedure [20]. A similar scheme of measurements was applied by Aoki et al. Authors reported that the Doppler examination of 70 patients subjected to ESWL also demonstrated a significant increase in the RI value directly after the procedure, in the ipsilateral kidney [21].

In the present study, RI and PI was reported to be a parameter of renal blood flow that can reflect changes in renal hemodynamics after RIRS. To my knowledge, the present study evaluated for the first time the variability of three intrarenal blood flow parameters after RIRS, with an assessment of the correlation between Doppler parameters and the mean stone volume, the energy used, and the effect of pre-stenting on the Doppler parameter changes. According to the results of this study, the mean value of RI and PI in the interlobar artery were significantly higher shortly after RIRS than before the procedure. RI and PI were significantly higher in the ipsilateral kidney than in the contralateral kidney just after RIRS. Sener et al. reported similar results in their study, that RI measured in the intrarenal artery (arcuate artery) increased significantly in the ipsilateral kidney after RIRS [13]. According to the study by Yazici et al., the mean RI of the arcuate arteries in the ipsilateral kidney increased significantly postoperatively [16]. The ipsilateral PI was also significantly higher than the contralateral kidney just after RIRS. Furthermore, Sener et al. reported that PI measurements in the arcuate artery were similar in the preoperative and postoperative periods without significant differences (the study was limited to the ipsilateral kidney only) [13]. Likewise, the acceleration time was not significantly different before and just after RIRS. The lack in changes of these Doppler parameters after RIRS allows the conclusion that AT is not a useful parameter for the evaluation of renal perfusion changes after RIRS. Due to the lack of studies published so far, this finding needs to be confirmed in further research. Balawender et al. evaluated the variability of AT among patients undergoing ESWL and found no significant differences in the values of this parameter after ESWL treatment [15]. In previous studies, the PI and RI values of the renal arteries in the preoperative and postoperative periods were similar and no statistically significant differences were reported. In my study, measurements were made only in the intrarenal arteries (on the level of the interlobar arteries). The most probable explanation for the lack of increased Doppler vascular resistance markers in the renal arteries is that the increase in intrarenal pressure does not directly affect the main trunks of the renal arteries, while the significant increase in resistance markers in the intrarenal vessels may be due to vasoconstriction of the intrarenal arteries, which may be related to the Venturi effect. Changes in the renin–angiotensin system may also explain the vascular changes observed during RIRS, with renal hypoperfusion due to high intrarenal pressure activating the renin–angiotensin system to increase renal perfusion as a potentially reflex mechanism. Increases in intrarenal pressure can also affect the contralateral kidney. Balawender et al. showed that vascular resistance parameters indicate a temporary impairment of perfusion after ESWL in both kidneys [20]. The pathomechanism of impaired perfusion in the kidney undergoing extracorporeal lithotripsy, and in the contralateral kidney, which remains outside of direct exposure to shock waves during the procedure, has not been fully elucidated. One theory identifies the formation of interstitial oedema around peripheral renal vascular branches as the cause of hemodynamic disturbance in the ipsilateral kidney [22]. The reflex release of substances that regulate the tension of the blood vessel wall (such as prostacyclins, nitric oxide, and endothelin) may also explain the blood supply disorders that occur in the contralateral. In this study, the postoperative RI of the interlobar artery of the contralateral kidney was similar to the preoperative values. In each regression model for the time point predictor before RIRS–just after RIRS, the RI and PI were significantly higher in the ipsilateral kidney than in the contralateral after RIRS. There were no significant differences in the acceleration time between the ipsilateral and contralateral kidneys just after RIRS.

Based on the analysis of regression models, the size of the stone undergoing laser lithotripsy and the value of the energy used are not factors that have a significant impact on the Doppler parameters during the RIRS procedure. An additional aspect of the study was to evaluate the effect of pre-stenting before RIRS on the value of measured Doppler parameters of renal blood flow after the procedure. Based on regression model analysis, the pre-stenting procedure has no significant effect on RI, PI, and AT after RIRS compared to the non-pre-stenting group. To my knowledge, it is the first prospective human study to evaluate the influence of pre-stenting on changes of intrarenal blood flow parameters after RIRS.

Doppler ultrasound examination was performed three times in each patient; hence, another aspect of the study is to compare the values of the measured parameters immediately after RIRS and 24 h later. The values of all three parameters 24 h after the procedure were comparable to their values immediately after the procedure. These results are particularly significant for the RI and PI values, demonstrating the persistence of a statistically significant elevated resistive index 24 h after surgery. The mean RI values at both time points (immediately after RIRS and 24 h later) did not exceed 0.7; therefore, from a clinical point of view, they do not represent risk factors for acute kidney injury. The evaluation of RI and PI values on consecutive days after RIRS requires further studies in the future.

The reported study has some limitations. First, monitoring of intrarenal pressure and intrarenal laser-induced heat production during the procedure was not available. To minimize this bias, standard saline irrigation at 50 cm with manual pumping was applied, which is the most commonly used irrigation system during RIRS. The increase in intrarenal temperature may have caused vasodilation of renal blood vessels. As we did not measure intrarenal heat during laser application, we were unable to assess the direct effect of increased intrarenal temperature on the renal vascular system. On the contrary, the procedure was performed using standard laser settings of 0.8–1 J energy and 8–10 Hz frequency (using these settings, the amount of intrarenal heat generated would be low).

The other limitation was the strict inclusion criteria for the study. The study cohort had no diseases or medications that could affect the vascular system. Therefore, the results cannot be extrapolated to the general population, which is dominated by diseases such as hypertension and diabetes that affect the vascular dynamics. Hence, the study cohort may not reflect the real world. Vascular changes during RIRS in such patient populations may differ and need to be evaluated in future studies. An additional limiting factor in eligibility for the study was the patient’s significant degree of obesity, due to the major difficulties in accurately visualizing the flow spectrum in the interlobar vessels. However, although this finding could not directly demonstrate an effect of intrarenal heat on the renal vascular system, it may indirectly be a finding related to this possible effect. The prospective design was a major strength of our study.

## 5. Conclusions

The statistically significant increase in RI and PI after RIRS in the ipsilateral kidney suggests a significant vasoconstriction of the interlobar arteries of the ipsilateral kidney generated by elevated intrarenal pressure during the procedure. The size of the stone exposed to laser lithotripsy, the value of the energy used, and pre-stenting are not factors that significantly influence Doppler parameters during RIRS (a summary of the most relevant findings of the study is provided in Table 4). Pending standardization of an intrarenal pressure monitoring system, Doppler evaluation is a noninvasive method of estimating intrarenal vascular perfusion changes during RIRS.

## Figures and Tables

**Table 1:** Sociodemographic data of the study sample, N_obs_ (the sample size) = 55.

Parameter	Distribution, Mdn (Q1–Q3)		*p*
	Women (*n* = 22)	Men (*n* = 34)	
Age	52.5 (44.5–62.0)	49.0 (42.0–57.0)	0.419
Weight	74.0 (64.0–82.8)	85.0 (78.0–89.0)	0.034
Height	164.0 (160.0–166.8)	176.0 (170.0–180.0)	<0.001
BMI	28.2 (24.6–31.9)	26.8 (25.11–29.4)	0.390

**Table 2:** Characteristics of clinical data without repeated measures, N_obs_ = 55.

Parameter	Measure	Distribution
Laterality:	-	
Right		27 (49.1%)
Left		28 (50.9%)
Pre-stenting:	-	
No		9 (16.3%)
Yes		46 (83.7%)
Mean stone volume	mm^3^	589.0 (426.0–706.5)
Energy used	kJ	6.8 (3.0–10.2)

**Table 3:** Characteristics of the clinical data with repeated measures, N_obs_ = 55.

Parameter	Kidney	Time	Distribution, Mdn (Q1–Q3)
Resistive index	ipsilateral	before RIRS	0.61 (0.56–0.66)
		0 h after RIRS	0.67 (0.63–0.73)
		24 h after RIRS	0.65 (0.60–0.70)
	contralateral	before RIRS	0.60 (0.56–0.64)
		0 h after RIRS	0.62 (0.58–0.65)
		24 h after RIRS	0.63 (0.58–0.68)
Pulsatility index	ipsilateral	before RIRS	1.06 (1.00–1.21)
		0 h after RIRS	1.20 (1.08–1.36)
		24 h after RIRS	1.20 (1.00–1.31)
	contralateral	before RIRS	1.01 (0.92–1.17)
		0 h after RIRS	1.05 (0.98–1.20)
		24 h after RIRS	1.08 (0.98–1.29)
Acceleration time	ipsilateral	before RIRS	25.90 (22.00–28.60)
		0 h after RIRS	27.60 (22.15–31.02)
		24 h after RIRS	27.60 (22.10–31.35)
	contralateral	before RIRS	25.30 (22.00–29.60)
		0 h after RIRS	27.60 (22.00–33.10)
		24 h after RIRS	26.30 (22.00–30.00)

**Table 4:** A summary of the results of the multivariate analysis including probability value.

Doppler Parameter	Time Period	*p*	Predictor	*p*
Resistive index	Before RIRS–0 h after RIRS		Mean stone volume	0.142
		0.010	Energy used	0.343
			Pre-stenting	0.096
Pulsatility index	Before RIRS–0 h after RIRS		Mean stone volume	0.170
		<0.010	Energy used	0.101
			Pre-stenting	0.194
Acceleration time	Before RIRS–0 h after RIRS		Mean stone volume	0.380
		0.206	Energy used	0.626
			Pre-stenting	0.240
Resistive index	0 h after RIRS–24 h after RIRS		Mean stone volume	0.261
		0.672	Energy used	0.484
			Pre-stenting	0.140
Pulsatility index	0 h after RIRS–24 h after RIRS		Mean stone volume	0.112
		0.116	Energy used	0.351
			Pre-stenting	0.398
Acceleration time	0 h after RIRS–24 h after RIRS		Mean stone volume	0.533
		0.057	Energy used	0.685
			Pre-stenting	0.312

## Data Availability

All the data is available within the study. This process can be initiated upon request to the corresponding author.
